# Current level of rheumatology teaching amongst undergraduate medical students: a systematic literature review

**DOI:** 10.1007/s10067-024-07297-5

**Published:** 2025-01-08

**Authors:** Koushan Kouranloo, Nikki Myall, Jennifer Christie

**Affiliations:** 1 Department of Rheumatology, Guy’s and St Thomas’ NHS Foundation Trust, London, UK; 2 School of Medicine, University of Liverpool, Cedar House, Ashton St., Liverpool, Merseyside L69 3GE UK; 3 British Medical Association (BMA) Library, Tavistock Square, London, WC1H 9JP UK; 4 Department of Rheumatology, Liverpool University NHS Foundation Trust, Mount Vernon, Liverpool, Merseyside L7 8YE UK

**Keywords:** Medical education, Rheumatology, Systematic review, Teaching, Undergraduate

## Abstract

Data from the British Society of Rheumatology demonstrate a lack of exposure to rheumatology for medical students, potentially impacting career choice. We conducted a Systematic Literature Review (SLR) on quantity, type and quality of rheumatology teaching for undergraduate medical students. This SLR was registered on PROSPERO (CRD42023472169). Articles published in English-language until February 2024 were included. Information was extracted on demographics, method(s) and hours for rheumatology teaching, and students’ and educators’ feedback, where reported. Eight cross-sectional studies were included, published between 1981 and 2024. Studies were conducted in UK (3); USA (2); Australia (1); Pan-European (1) and Uganda (1). Year of rheumatology teaching at medical school was reported in four studies (three UK; one USA). The three UK studies taught rheumatology in the latter years of study; in the USA study, rheumatology was taught every year. Duration of exposure to rheumatology was 15–96 h per student. Reported teaching methods included lecture-based, rheumatology inpatient service, shadowing consultations and patient education. Student feedback was available in one (UK based) study- four students felt they had limited exposure to rheumatology; eight considered it “niche”. Factors identified for poor exposure to rheumatology included lack of full-time rheumatologists on the school’s faculty; lack of specialty training programmes in local hospitals; greater emphasis on acute specialties (e.g. emergency medicine). Exposure to and student awareness of clinical rheumatology has markedly decreased over time. Given the ageing population and increase in multimorbidity, there is a need to increase exposure and encourage entry to rheumatology training.

**Table:** 

**Key Points** • *There are clear differences between the methods of rheumatology teaching between institutions and countries.* • *Teaching hours dedicated to undergraduate rheumatology education have overall markedly decreased over time, resulting in variable student awareness of the depth and breadth of this speciality.* • *The overall lack of exposure to rheumatology in undergraduate programmes has been associated with a lack of uptake of rheumatology as a career amongst medical students globally.* • *Our results indicate a need for a minimum required number of hours of teaching dedicated to rheumatology, delivered by specialists as well as a standardised undergraduate curriculum of competencies to better reflect the needs of our increasingly ageing and multimorbid population.*

**Supplementary Information:**

The online version contains supplementary material available at 10.1007/s10067-024-07297-5.

## Introduction

Since Modernising Medical Careers (MMC) in the United Kingdom (UK) in 2007, newly qualified doctors are expected to choose their specialty of choice much sooner than previously [1]. Since this change, new graduates from medical schools must now enter a structured 2-year training programme known as Foundation Programme (FP), usually including six 4-month placements in a wide range of specialities, which can include (but are not limited to) medical and surgical specialties, General Practice, Intensive Care, paediatrics and psychiatry [2, 3]. This allocation is currently carried out as per a Score Based Allocation based on their Education Performance Measure (EPM) during medical school plus a Situational Judgement Test (SJT) [3]. During the second year of FP, approximately 18 months after graduating from medical school, Foundation Doctors are expected to decide on and apply to a speciality of choice. This contrasts with the arrangement in place prior to MMC, when new medical graduates often had the opportunity to work in different specialities over a period of years prior to being expected to make a career decision. Due to these changes, young doctors have frequently had less experience to decide on a lifelong career prior to applying to their chosen specialty, with some of the applicants applying to specialities without any formal experience or time spent in those specialities during their FPs. Currently in the UK with approximately 65 different specialities to choose from after successful completion of FP, the process of choosing a career of choice can be challenging for many junior doctors [4].

As per the latest British Society for Rheumatology (BSR) report in 2021, there appears to be chronic workforce shortages to provide safe staffing in rheumatology in the UK, with a deficit of consultant rheumatologists across the country. These workforce shortages lead to poorer health outcomes for patients, particularly in rural areas away from large cities, which also corresponds with larger consultant vacancies [5]. Furthermore, it is estimated that 360 rheumatology consultants are likely to retire within the next 5 years across the UK, which will exacerbate this crisis. This lack of consultant role models in the specialty is likely to contribute to lack of exposure for the medical students and Foundation Doctors.

It is well documented that the experience of medical students on placement in various specialties during medical school is crucial in determining their future career choice [6–9]. Therefore, understanding the factors which influence medical students’ perceptions about different specialities remain the key to better understanding their potential future choices This can then help individual medical school to equip their curriculum to increase exposure of medical students to less-desired, yet fulfilling potential future career options, which could have a subsequent effect on the workforce planning and future recruitment in those specialties. This coupled with establishing the current teaching practices of rheumatology delivered across different countries (including the UK), can help with shaping the future of rheumatology training while addressing our population needs.

We conducted a systematic literature review (SLR) to determine the quantity, type and experience of rheumatology teaching received at medical schools globally.

## Methods

This SLR was conducted with accordance to the Cochrane Handbook and reported as per the Preferred Reporting Items for Systematic Reviews and Meta-Analysis.

The protocol was developed and registered in the PROSPERO database of Systematic Reviews (CRD42023472169). The review question was what is the current level of rheumatology teaching amongst UG medical students.

### Population

The population was defined as the rheumatology teaching received by UG medical students.

### Outcome

The main outcomes were the duration and type of UG rheumatology training received by medical students. There were no restrictions on institution, region or country.

The main outcomes were the duration, type and frequency of curricular teaching receiving on rheumatology by UG medical students.

### Search strategy, databases and study selection

The search strategies are available in the online supplementary material. To ensure full comprehensive coverage, indexing terms (Medical Subject Heading-MeSH, applicable to Medline and Cochrane and Emtree headings used on Embase) along with relevant keywords were incorporated. Medline, Embase and Cochrane were searched from 1946 until June 2023, restricted to English-language articles only. Relevant websites for national bodies involved in and overseeing rheumatology education were also searched separately. Eligible articles were observational studies, trials, case series of 10 or more. Case reports, case series of less than 10, opinion articles, editorials, literature reviews and studies not including UG medical students were excluded.

We excluded studies not including UG medical students and those studies discussing a single teaching intervention on a select cohort rather than sustained curricular teaching activity.

Information was extracted on the type, length and frequency of rheumatology teaching received, including the year and the setting. Students’ and educators’ feedback was gathered when mentioned.

Titles and abstracts were screened by KK, to assess eligibility. The full articles which met the inclusion criteria were then examined in detail by KK. For validation, 20% of the articles were screened at the abstract and full paper stage by a second author JC.

## Results

**Fig. 1:**
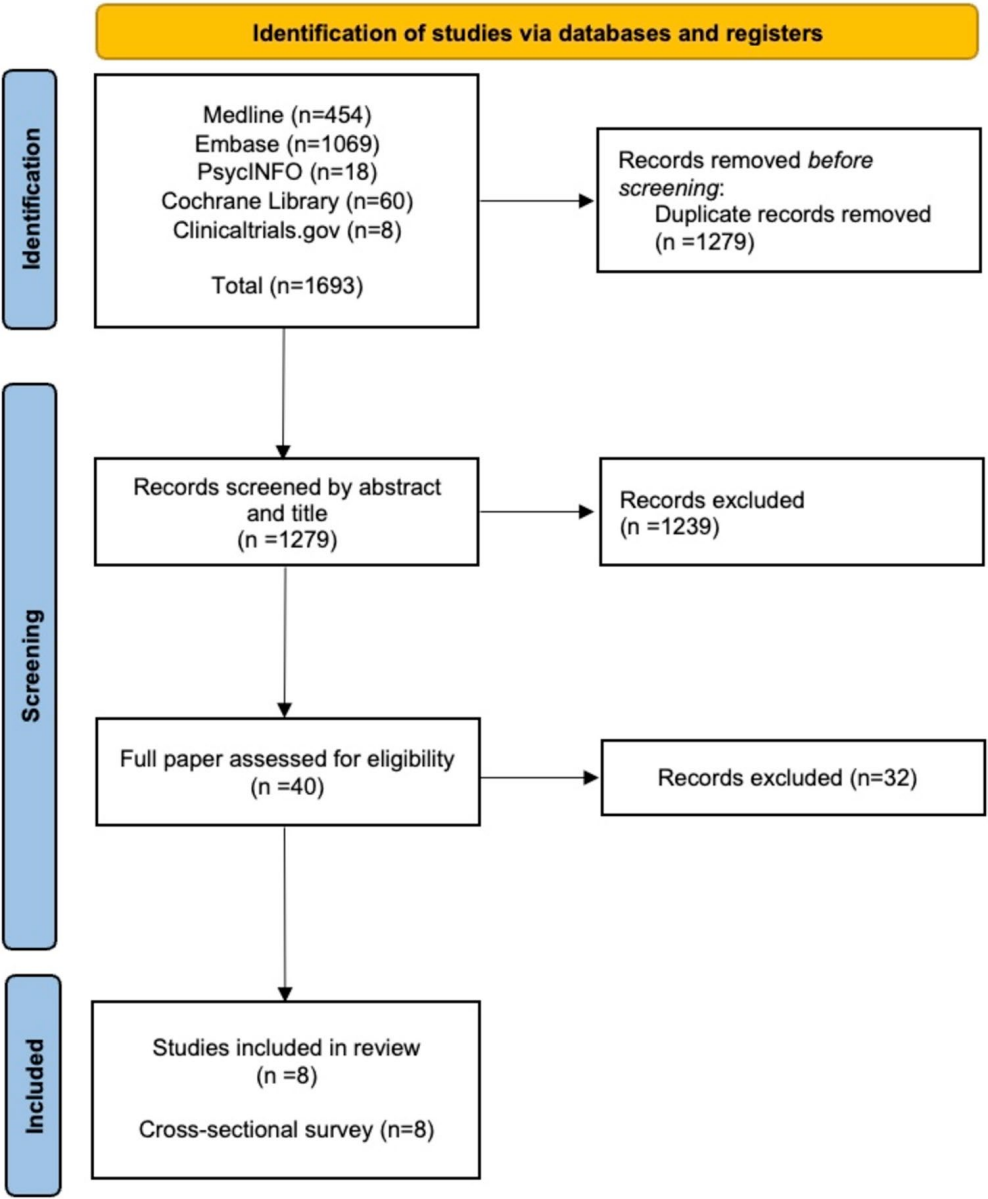
PRISMA flowchart of included papers

After deduplication, 1195 articles were retrieved. After screening title and abstract, 1156 papers were excluded, with 39 proceeding to full-text screening. Ultimately, eight articles (all cross-sectional surveys) were included (Fig. 1; Table 1). Publication year of included articles ranged from 1981 to 2018. The countries of included studies were UK (3), USA (2), Australian (1), European (1) and Uganda (1) [10–17].

**Table 1:** Summary of included studies. *UK*, United Kingdom; *USA*, United States of America

Author	Country	Population/setting	Sample size (schools and/or students)	Duration of study	Methodology	Time (hours)	Summary of author conclusions
Abhishek et al. 2018 [10]	Pan-Europe	Medical schools across Europe	21 medical schools across 11 European countries	Survey sent in January 2017 with follow-up reminder	Survey questionnaire	N/A	Survey identified areas of similarities and differences in the undergraduate rheumatology curriculum across Europe; suggests the development of a core European curriculum
DiGiovanni et al. 2016 [11]	USA	136 USA medical schools for the 2014–2015 academic year	136 medical school websites	Academic year 2014–2015	Website analysis	80 ± 40	Musculoskeletal medicine is underrepresented in USA medical school clinical curriculum, highlighting need for curricular reform
Goldenberg et al. 1981 [12]	USA	USA Medical Schools	119 medical schools	Not specified	Questionnaire sent to Rheumatology Program Directors at USA medical schools	4–8	Rheumatology education in USA medical schools is varied; some schools lack sufficient resources and faculty for comprehensive training
Jandial et al. 2009 [13]	UK	UK Medical Schools	23 UK medical schools	Not Specified	Structured questionnaires sent to child health leads at UK medical schools	N/A	pMSK clinical teaching not core to most UK medical schools, with variable content and delivery; infrequently included in assessments. It is important that pMSK subspecialities such as rheumatologists and paediatric orthopedic surgeons are involved in the curriculum design of pMSK but this is not currently common place, presumably because they are only available at larger tertiary centres and not in peripheral teaching hospitals
Kay et al. 2000 [14]	UK	UK Medical Schools	23 UK medical schools	Not specified	Questionnaire sent to lead rheumatology teachers at UK medical schools	69	Variability in rheumatology teaching across UK medical schools; need for more structured and consistent curriculum
McColl et al. 2005 [15]	Asia–Pacific	130 medical schools in Asia–Pacific region; 44 responses received	44 medical schools	Not specified	Questionnaire sent to medical schools	69.7	Musculoskeletal teaching in Asia–Pacific medical schools is comparable to other regions; lacks in certain areas, e.g. pain management and metabolic bone disease
Thapper et al. 2013 [16]	UK	11 UK medical schools	256 students	2 months	Online questionnaire distributed to fourth-, fifth- and sixth-year medical students to all 31 UK medical schools	0–120	Medical students in the UK have varied attitudes towards rheumatology training; exposure to rheumatology influences interest in the specialty
Kibone et al. 2024 [17]	Uganda	9 medical institutions	359 students	Survey conducted March–April 2022	Self-administered semi-structured questionnaire	N/A	Authors found a significant gap in knowledge on rheumatology and RMDs amongst medical students in Uganda. Need for interventions to increase contact time between students and rheumatologists as well as patients living with RMDs to improve their knowledge

Year of rheumatology teaching at medical school was reported in four studies (3 UK and 1 USA) [12–14, 16]. The three UK studies were all taught in the latter years [4–6], whereas in the study based in the USA, the students were taught rheumatology in every year. Only one UK-based study, including 23 out of 30 British medical schools, looked specifically at paediatric rheumatology under the heading of “Musculoskeletal (MSK)”. This article reported that paediatric rheumatology is done mostly primarily in later years of undergraduate course with year 4 (15/23) and year 5 (10/23), with less exposure in earlier years with year 1 (1/23), year 2 (2/23) and year 3 (7/23) [13].

Where reported, data on the number of hours of exposure to rheumatology were variable, ranging from 4 to 120 h per medical student. Noticeably, in a 2013 UK based study by Thapper et al. on a national survey of undergraduate medical students in 4th, 5th and 6th year of their studies, approximately 1/5 (20.7) of the total amount received no rheumatology teaching [16].

Reported methods of rheumatology teaching included lecture-based, problem-based learning (PBL), rheumatology inpatient service (i.e. bedside), shadowing consultations, elective/selected modules and with patient educators.

Student feedback was provided in two studies, one based in the UK and one in Uganda. In the UK-based study, which surveyed 49 students, two regarded rheumatology as “fascinating”, four felt that they had limited exposure to the specialty and eight considered it as “a niche specialty of no interest”. In the Ugandan study, 15% of 359 students thought rheumatology is solely the study of “rheumatic fever and/or rheumatic heart disease” [16, 17].

No feedback from educators were collected in any of the included studies. Where mentioned these educators ranged from rheumatology consultants (staff), physicians, registrars (fellows), residents, specialist nurses and patient educators. One UK-based study by Kay et al. specifically mentioned the use of American College of Rheumatology (ARC) educational resources to aid with teaching [14].

Factors identified by the included studies for overall poor exposure to rheumatology included lack of having full-time rheumatologists on the school’s faculty, lack of specialty training programme in their local teaching hospital as the result of other curriculum pressures and greater emphasis on general medical/acute specialties (e.g. general practice and emergency medicine).

## Discussion

To our knowledge, this is the first SLR summarising the type, length and quality of rheumatology teaching at UG level. Despite the relatively small number of studies included in this SLR, we have managed a to capture a good international representation of rheumatology exposure and teaching during undergraduate medical training. However, there seems to be significant variation in delivery of rheumatological topics at UG level, even in countries geographically close to each other and amongst medical schools in the same country. For instance, as per the survey done by Abishek et al. comprising 11 European countries, differentiating between mechanical versus inflammatory joint pain was thought in nearly all of them, whereas only a few medical schools taught about different disease-modifying drugs (DMARDs) or the long-term physical or psychological effects of having a chronic autoimmune condition [10]. Addressing these discrepancies and ensuring appropriate inclusion of both physical and mental health consequences of chronic disease, in conjunction with other comorbidities, remain key for countries like the UK that are dealing with an ever-increasing rate of polypharmacy in a multimorbid ageing population.

Musculoskeletal disorders are one of the most common presentations to primary care in many countries including the UK [18, 19]. However, only a limited number of curriculum time is allocated to teaching these at UG level. This is also true in a similar Canadian based study, where MSK complaints counts for 13.7–27.8% of primary care time, despite having only 2.3% of dedicated curriculum time for medical students [20]. Therefore, appropriately educating medical students to confidently deal with MSK conditions remains essential. Rheumatology remains one of the main specialities in delivering teaching of MSK disorders. However, education in MSK disorders at UG level is commonly placed between rheumatology, trauma and orthopaedics (T&O) as well as General Practice (GP)/primary care. Based on our results, the extent to which this teaching is delivered by each of these specialities depends on the local postgraduate training facilities and rheumatology staff, the availability of these services in the community as well as other members of the multidisciplinary team (MDT) such as specialist nurses or physiotherapists.

Based on our results, where mentioned, the specific rheumatology teaching hours had a wide range between 4 and 120 h per medical student. In a survey of American medical schools by DiGiovonni et al., it was reported that MSK disorders were taught by rheumatologists only by one American medical school, despite it being a common presenting complaint to the practicing rheumatologists [11]. This lack of exposure to rheumatologists results in UG students having less overall exposure to common rheumatological conditions such as inflammatory arthritis (with more of a focus on non-inflammatory conditions such as mechanical joint pain), as well as to the speciality as a whole, undoubtedly subsequently impacting future career choice.

This lack of rheumatology representation was also noted in a survey on Asia–Pacific medical schools carried out by Mccol et al., who reported that the number of teaching hours devoted to orthopaedics was double that of rheumatology. This was attributed to the variation in practice of management of MSK disorders across the Asia–Pacific countries and that orthopaedics services are frequently in older and larger established institutions when compared with rheumatology faculties [15].

With regard to career choice, as demonstrated by Dunckley et al., specialist registrars (SpRs) choose their speciality choice based on exposures during what was previously known as Senior House Officer (SHO) years prior to MMC in 2007 [1, 21, 22]. It may be suggested that after the introduction of MMC, exposure to different specialities during UG years leaves a large influence on medical students deciding on their selected specialty over the 2 years of Foundation Training. In a UK-based survey by Rossoue et al. of senior (4th, 5th and 6th years) medical students, most students had received 3 weeks (96 h) exposure to rheumatology while 20.7% of respondents had received no teaching on rheumatology [16]. Importantly, Rossoue et al. demonstrated that at the time of the study, only 154 Foundation Programme jobs across the UK had dedicated rheumatology placement out of a total of 4970. One hundred fifteen out of one hundred fifty-four of these rheumatology jobs were across the four deaneries of London [16]. These limited, mostly London-based posts, not only deprive those students interested in further experiencing and developing rheumatology-specific skills during their FP, but also could explain the current rheumatology recruitment crisis as demonstrated by BSR, especially outside major cities.

It is also noteworthy that as of 2025, the current Score Based Allocation of Foundation Programme jobs is going to be radically changed to Preference Informed Allocation, where the previously discussed Educational Performance Measure (EPM) and Situational Judgement Test (SJT) are going to be removed and the medical students are going to be asked to rank Foundation Schools based on their order of preference [3]. Once they have been allocated to a Foundation School, their specific NHS Trust allocation within each region is still going to be done at random and via a computer-generated algorithm. While this could on face value be considered a potential solution to diversifying the career choices of recent graduates, the lack of established rheumatology departments or allocated rheumatology FP placements in smaller, more rural centres will only be detrimental in trying to hone enthusiasm towards the specialty and encourage applications at specialty training level.

The small number of rheumatology posts relative to the larger medical specialties will limit FP doctors’ exposure to a thorough understanding of rheumatology as a speciality and results in fewer rheumatology-related educational opportunities, making them “less competitive” to apply to rheumatology as a competitive speciality of choice in Postgraduate medicine. Of note, rheumatology specialty training applications had a competition ratio of 1 in 6.03 in year 2020 [23]. This competition ratio of 1:6 would suggest that there is clearly not a lack of applicants to the specialty but rather a lack of quality and experience which both could be explained by lack of previous exposure to the speciality as an undergraduate or Foundation Trainee. This is in addition to an estimate of 15–20% of time of rheumatology registrars have been lost to acute general medicine since the introduction of Internal Medicine Year 3 (IMT3) year (5).

## Future directions

Based on the studies retrieved, there are limited data available on undergraduate medical students’ perceptions towards rheumatology as a future potential career, particularly outside of North America and Europe. Various factors have been identified which influence popularity of given specialties amongst medical students and postgraduate doctors in different countries. These range from their experiences during training, to factors such as work-life balance, monetary remuneration and influence of potential mentors. However, at the heart of all of these is the requirement for adequate exposure to the specialty throughout their training. Certain specialities may be perceived as “unpopular”, and this will undoubtedly vary from region to region globally. However, as demonstrated through this SLR, for rheumatology as a specialty, this seems to be due to lack of awareness of what the specialty entails, and this is rooted right from undergraduate level. Strategies to increase awareness, at both local and national levels, may involve organisations such as British Society for Rheumatology and Royal College of Physicians.

Furthermore, more research is needed to better understand the perception amongst medical students of those specialties which have historically struggled to recruit. This in itself could highlight specific areas to nurture or develop further to increase the attractiveness of these specialties amongst future undergraduate students. Our data show that only one study gathered feedback from educators, and therefore, there is an apparent lack of information on the teaching of rheumatology from the trainers’ perspective. Future research in this area would need to be multi-centre, given the heterogeneity of curricula even within a single country, to better capture the current school of thought amongst students as well as their trainers on rheumatology as a career. This would aim to ensure that data on current methods can be effectively captured and synthesised, subsequently leading to large-scale change.

## Strength and limitations

To our knowledge, this is the first SLR on the type, length and experience of rheumatology teaching amongst UG students globally with good international representations despite the relative few suitable studies. However, all of these studies are cross-sectional surveys and they always potentially have the risk of non-response bias. Therefore, the results reported here may not be the actual representation of the current level of rheumatology teaching at the undergraduate level. Furthermore, due to the nature of these studies, any additional change over time towards the UG rheumatology curriculum has not been made able to include.

Furthermore, although we tried to take some indirect conclusion on the quality if the rheumatology teaching from our included studies through students’ and clinicians’ experience, true assessment of quality of teaching remains a multifaceted phenomenon which in most cases requires analysis of results in long term, which is not possible with cross-sectional survey studies.

## Conclusion

Overall, despite variations between and within countries with regards to the delivery of teaching, there seems to be limited dedicated hours to rheumatology teaching to undergraduate medical students, which has directly markedly decreased the exposure to clinical rheumatology over time, resulting in variable student awareness of the depth and breadth of this speciality. This can have a direct impact on the “popularity” of rheumatology as a career of choice amongst medical students globally leading to the recruitment crisis currently seen at consultant levels in the both the UK and USA. This is also not mirroring the well-known, yet increasing, number of MSK complaints in primary care in these countries coupled with the demands of an ageing, multimorbid populations. Orchestrated efforts are needed to not only introduce a minimum hour of rheumatology-specific teaching delivered by the those in the specialty, but also introduce a national undergraduate curriculum of competencies to better reflect the needs of our current population.

## Supplementary Information

Below is the link to the electronic supplementary material.

Supplementary file1 (DOCX 18 KB)

## Data Availability

All data available upon reasonable request.
