# Supplementary material for: Current level of rheumatology teaching amongst undergraduate medical students: a systematic literature review
Source: Clin Rheumatol. 2025 Jan 8;44(2):537–45. doi: 10.1007/s10067-024-07297-5 (PMC11774963; doi:10.1007/s10067-024-07297-5)
Supplement: Supplementary file 1 — Supplementary file1 (DOCX 18 KB) [file 10067_2024_7297_MOESM1_ESM.docx]

**Supplementary material: Search Strategies**

**A.1 Medline**

| 1 | Students, Medical/ or Education, Medical, Undergraduate/ |
| --- | --- |
| 2 | ((medicine or medical) adj2 (undergrad$ or student$ or educat$ or school$ or studying)).ti,ab,kw,kf. |
| 3 | 1 or 2 |
| 4 | Rheumatology/ |
| 5 | rheumatolog$.ti,ab,kw,kf. |
| 6 | 4 or 5 |
| 7 | 3 and 6 |
| 8 | Schools, Medical/ or Teaching/ or Curriculum/ or Career Choice/ |
| 9 | (training or trainee$ or educat$ or curricul$ or module$ or teach$ or taught or learn$ or program$ or speciality or specialities or specialise or future career$ or career choice or recruitment).ti,ab,kw,kf. |
| 10 | ed.fs. |
| 11 | or/8-10 |
| 12 | 7 and 11 |
| 13 | limit 12 to english language |

A.2 Embase

| 1 | medical student/ or undergraduate education/ |
| --- | --- |
| 2 | ((medicine or medical) adj2 (undergrad$ or student$ or educat$ or school$ or studying)).ti,ab,kw,kf. |
| 3 | 1 or 2 |
| 4 | rheumatology/ |
| 5 | rheumatolog$.ti,ab,kw,kf. |
| 6 | 4 or 5 |
| 7 | 3 and 6 |
| 8 | medical school/ or *teaching/ or curriculum development/ or career/ |
| 9 | (training or trainee$ or educat$ or curricul$ or module$ or teach$ or taught or learn$ or program$ or speciality or specialities or specialise or future career$ or career choice or recruitment).ti,ab,kw,kf. |
| 10 | or/8-9 |
| 11 | 7 and 10 |
| 12 | limit 11 to english language |

**A.3 PsycINFO**

| 1 | medical students/ or undergraduate education/ |
| --- | --- |
| 2 | ((medicine or medical) adj2 (undergrad$ or student$ or educat$ or school$ or studying)).ti,ab,tw. |
| 3 | 1 or 2 |
| 4 | Rheumatoid Arthritis/ |
| 5 | rheumatolog$.ti,ab,tw. |
| 6 | 4 or 5 |
| 7 | 3 and 6 |
| 8 | exp teaching/ or curriculum development/ or occupational choice/ |
| 9 | (training or trainee$ or educat$ or curricul$ or module$ or teach$ or taught or learn$ or program$ or speciality or specialities or specialise or future career$ or career choice or recruitment).ti,ab,tw. |
| 10 | 8 or 9 |
| 11 | 7 and 10 |
| 12 | limit 11 to english language |

**A.4 Cochrane**

| #1 | MeSH descriptor: [Students, Medical] this term only |
| --- | --- |
| #2 | MeSH descriptor: [Education, Medical, Undergraduate] this term only |
| #3 | ((medicine or medical) NEAR/2 (undergrad* or student* or educat* or school* or studying)):ti,ab,kw |
| #4 | #1 OR #2 OR #3 |
| #5 | MeSH descriptor: [Rheumatology] this term only |
| #6 | (Rheumatolog*):ti,ab,kw |
| #7 | #5 OR #6 |
| #8 | #4 AND #7 |
| #9 | MeSH descriptor: [Schools, Medical] this term only |
| #10 | MeSH descriptor: [Teaching] this term only |
| #11 | MeSH descriptor: [Curriculum] this term only |
| #12 | MeSH descriptor: [Career Choice] this term only |
| #13 | (training or trainee* or educat* or curricul* or module* or teach* or taught or learn* or program* or speciality or specialities or specialise or future NEXT career* or "career choice" or recruitment):ti,ab,kw |
| #14 | #9 OR #10 OR #11 OR #12 OR #13 |
| #15 | #8 AND #14 |

**A.5 Clinicaltrials.gov**

rheumatology AND (teaching OR career) AND (student OR undergraduate)

**A.6 WHO International Clinical Trials Registry Platform (ICTRP)**

rheumatology AND (teaching OR career) AND (student OR undergraduate)

**A.7 Google Scholar**

allintext:rheumatology AND (curriculum OR career) AND ("medical student" OR "medical undergraduate")

**A.8 General Medical Council (GMC)**

Rheumatology

<https://www.gmc-uk.org/searchresults?searchText=rheumatology&page=1>

**A.9 British Society for Rheumatology (BSR)**

(teaching OR curriculum OR training) site:rheumatology.org.uk

**A.10 American College of Rheumatology (ACR)**

(teaching OR curriculum OR training)

**A.11 European League Against Rheumatism (EULAR)**

(teaching OR curriculum OR training) students site:eular.org

**A.12 Association for the Study of Medical Education (ASME)**

Rheumatology or rheumatic
